# Development of Carvedilol Nanoformulation-Loaded Poloxamer-Based In Situ Gel for the Management of Glaucoma

**DOI:** 10.3390/gels9120952

**Published:** 2023-12-04

**Authors:** Bjad K. Almutairy, El-Sayed Khafagy, Amr Selim Abu Lila

**Affiliations:** 1 Department of Pharmaceutics, College of Pharmacy, Prince Sattam Bin Abdulaziz University, Al-Kharj 11942, Saudi Arabia; b.almutairy@psau.edu.sa; 2 Department of Pharmaceutics and Industrial Pharmacy, Faculty of Pharmacy, Suez Canal University, Ismailia 41522, Egypt; 3 Department of Pharmaceutics and Industrial Pharmacy, Faculty of Pharmacy, Zagazig University, Zagazig 44519, Egypt; a.abulila@uoh.edu.sa; 4 Department of Pharmaceutics, College of Pharmacy, University of Hail, Hail 81442, Saudi Arabia; 5 Medical and Diagnostic Research Center, University of Hail, Hail 81442, Saudi Arabia

**Keywords:** carvedilol, glaucoma, in situ gel, poloxamer, spanlastics

## Abstract

The objective of the current study was to fabricate a thermosensitive in situ gelling system for the ocular delivery of carvedilol-loaded spanlastics (CRV-SPLs). In situ gel formulations were prepared using poloxamer analogs by a cold method and was further laden with carvedilol-loaded spanlastics to boost the precorneal retention of the drug. The gelation capacity, rheological characteristics, muco-adhesion force and in vitro release of various in situ gel formulations (CS-ISGs) were studied. The optimized formula (F2) obtained at 22% *w*/*v* poloxamer 407 and 5% *w*/*v* poloxamer 188 was found to have good gelation capacity at body temperature with acceptable muco-adhesion properties, appropriate viscosity at 25 °C that would ease its ocular application, and relatively higher viscosity at 37 °C that promoted prolonged ocular residence of the formulation post eye instillation and displayed a sustained in vitro drug release pattern. Ex vivo transcorneal penetration studies through excised rabbit cornea revealed that F2 elicited a remarkable (*p* ˂ 0.05) improvement in CRV apparent permeation coefficient (P_app_ = 6.39 × 10^−6^ cm/s) compared to plain carvedilol-loaded in situ gel (CRV-ISG; P_app_ = 2.67 × 10^−6^ cm/s). Most importantly, in normal rabbits, the optimized formula (F2) resulted in a sustained intraocular pressure reduction and a significant enhancement in the ocular bioavailability of carvedilol, as manifested by a 2-fold increase in the AUC_0–6h_ of CRV in the aqueous humor, compared to plain CRV-ISG formulation. To sum up, the developed thermosensitive in situ gelling system might represent a plausible carrier for ophthalmic drug delivery for better management of glaucoma.

## 1. Introduction

Glaucoma is a neurodegenerative disorder that is characterized by progressive optic nerve degeneration, leading to permanent blindness [1]. Elevated intraocular pressure (IOP) stands as a major risk factor for developing glaucoma. According to the World Health Organization (WHO), glaucoma is considered the second leading cause of visual impairment and blindness in the world [2]. Medical treatment of glaucoma includes topical beta-adrenergic blockers (timolol, betaxolol and metipranolol), alpha agonists (Brimonidine), carbonic anhydrase inhibitors (methazolamide and acetazolamide), prostaglandin analogs (latanoprost, bimatoprost and travoprost), and rho kinase inhibitors (netarsudil and ripasudil) [3]. Carvedilol is a beta-adrenergic blocker that is used to treat hypertension and ischemic heart diseases. Carvedilol is a class II (high permeability/low solubility) drug according to Biopharmaceutical Classification System. It shows poor bioavailability (~25%); owing to extensive first-pass metabolism, following oral administration [4]. Nevertheless, recent research has underscored the plausible use of topical carvedilol to treat high intraocular pressure [5,6]. For instance, Hassan et al. have affirmed the efficacy of ocularly applied carvedilol-loaded cationic nanoparticle (leciplex) in reducing the intraocular pressure to a normal range in ocular hypertensive rabbits [6].

A major challenge in ocular drug delivery is to tailor a delivery system that can grant adequate drug concentrations at the target region for sufficient time interval. Conventional ocular drug delivery systems, such as eye drops and ointments, usually suffer from poor drug bioavailability, presumably, due to the complex anatomy and highly selective physiological corneal barriers, which hinder the entry of exogenous materials to the ocular tissues [7]. In addition, excessive and rapid loss of drug due to high tear fluid turnover and the nasolacrimal drainage could reduce ocular absorption by reducing the contact time of instilled drug at the site of action [8].

Colloidal drug delivery systems such as nanoparticles, nanoemulsions, niosomes, and liposomes have been extensively explored in ocular drug delivery [9,10,11]. The benefits of colloidal carriers include controlled and/or sustained drug release at the targeted site, the ability to deliver both hydrophilic and hydrophobic drugs to eye tissue, and the potential to circumvent blood-ocular barriers/efflux-related problems encountered with the parent drug [10,11]. Among a wide range of colloidal carriers in the realm of ocular drug delivery, spanlastics (SLs), an elastic non-ionic surfactant based vesicular system, have emerged as a viable delivery vehicle that could efficiently circumvent the limitations of conventional ophthalmic drug delivery systems [12,13]. Spanlastics are composed of a non-ionic surfactant as a main vesicle forming component, and an edge activator (EA). The inclusion of EA to vesicles provides SLs with a great elasticity, compared to conventional niosomes [14]. Such elasticity of the vesicles enhances the corneal permeability of the entrapped drugs, underscoring the potential use of SLs as efficient drug delivery vehicles for ocular administration [15].

In situ gelling systems are stimuli-sensitive polymeric viscous liquids that undergo sol to gel transformation upon application to the human body in response to small changes in specific conditions like pH, temperature and/or ionic strength [16]. Recently, ophthalmic in situ gels have emerged as an ideal choice for ocular delivery. Compared to conventional gels, in situ gels are applied as solutions or suspensions, which provides ease of administration while maintaining dose accuracy. Afterwards, they are converted into a gel state upon contact with tear fluids in response to definite stimuli such as temperature, pH, etc. [17]. This would extend ocular residence time, decrease pre-corneal elimination, and consequently, enhance the ocular availability of the administered drug along with improving patient compliance via reducing dosing frequency [18]. Rawat et al. [8] have recently emphasized the efficacy of dual-responsive in situ gel, composed of a combination of the thermo-sensitive poloxamers (poloxamer 407/poloxamer 188) and the ion-sensitive polymer kappa-carrageenan, for enhancing the anti-glaucoma potential of the β-adrenergic antagonist, nebivolol. Intriguingly, the ocular applicability of in situ gels can be augmented by the incorporation of nanoparticulate systems within in situ gel with the goal of extending drug release and enhancing the therapeutic outcomes [19,20]. 

This study, therefore, aimed at formulating carvedilol in a dosage form, spanlastic-laden in situ gel system, that combine the advantages of both nano-systems (spanlastics) and in situ gels as a plausible tool to sustain drug delivery, enhance drug transcorneal permeation and eventually ameliorate its ocular bioavailability for the management of glaucoma. For such purpose, thermosensitive polymers; poloxamer 407/poloxamer 188, were utilized for the formulation of in situ gel system. Formulation parameters such as gelation temperature, muco-adhesion force and in vitro release behavior of in situ gel systems were optimized. Finally, the ex-vivo permeation, the in vivo fate, and the in vivo efficacy of optimized carvedilol-loaded in situ gel system in lowering IOP were investigated.

## 2. Results and Discussion

### 2.1. Formulation of Carvedilol-Loaded Spanlastic In Situ Gel (CS-ISG)

In our previous study, we succeeded to formulate carvedilol-loaded spanlastics (CRV-SPLs) for augmenting the therapeutic efficacy of carvedilol in a hypertensive rat model [21]. In that study, a combination of Span 60 as main vesicle component and different edge activators (EA), namely Tween 80 and Brij 97, were adopted for the fabrication of CRV-loaded spanlastics at two Span:EA ratios (90:10 and 80:20). It was evident that spanlastics prepared with Brij 97 as an edge activator at a Span:EA ratio of 80:20 showed optimal formulation attributes such as minimum vesicle diameter, high entrapment efficiency, and efficient drug permeability. In this study, we tried to extend our work via challenging the efficacy of CRV-loaded spanlastics (CRV-SPLs) in the management of glaucoma. For such purpose, the optimized CRV-loaded spanlastic formulation (CRV-SPLs) was incorporated into an in situ gel formulation (CS-ISG) to facilitate its application, extend the residence time onto the corneal surface after application, and subsequently, augmenting drug penetration through the cornea. Herein, poloxamer-based thermo-sensitive polymers (poloxamer 407/poloxamer 188) were utilized for the fabrication of carvedilol-loaded spanlastic in situ gel (CS-ISG) formulations. Poloxamer-based polymers were selected because of their advantages of high-water solubility, thermo-reversible gelation properties, and their ability to produce translucent gels that do not interfere with normal vision. The composition of different poloxamer-based in situ gel formulation is summarized in Table 1.

### 2.2. Evaluation of CS-ISGs

#### 2.2.1. Clarity 

Clarity and homogeneity of various CS-ISG formulations were assessed visually before and after loading with CRV-SPLs. Plain ISGs were transparent and homogeneous; however, following CRV-SPLs loading, CS-ISGs became less transparent, but with no suspended particles. 

#### 2.2.2. pH

Generally, for ophthalmic products to be well tolerated by eyes, the pH of the fabricated products should fall within the normal ocular comfort range (pH range of 6.5 to 7.5) [22]. As illustrated in Table 2, the pH of all CS-ISG formulations was determined to be between 6.48 ± 0.04 and 7.32 ± 0.05, which is considered within the acceptable limit for ophthalmic treatments. 

#### 2.2.3. Drug Content

All the prepared CS-ISG formulations showed high drug content fluctuating from 97.3 ± 0.9% to 99.3 ± 0.4% (Table 2). These results suggest that CRV-SPLs were uniformly distributed within the prepared ISGs, and the preparation method was reproducible. 

#### 2.2.4. Gelation Temperature

Gelation temperature (T_G_) is an important metric in determining the ability of the applied ISG formulation to be transformed into gel state at ocular temperature upon eye installation. To be readily instilled into the eye, an ophthalmic in situ gel should have a T_G_ greater than room temperature (25 °C) and be transformed into a gel at pre-corneal temperature (35 °C). Formulation with T_G_ greater than 37 °C is not desirable for ophthalmic use because these formulations would stay in the sol state after ocular administration and could suffer from nasolacrimal drainage prior exhibiting its pharmacological effect. In this study, T_G_ of various CS-ISG formulations ranged from 28.8 ± 0.6 °C (F6) to 40.5 ± 0.8 °C (F7) (Table 2). It was obvious that, at fixed P188 concentration, increasing P407 concentrations from 20% *w*/*v* to 25% *w*/*v* results in a significant decrease in gelation temperature. For instant, in situ gel formula prepared at 25% *w*/*v* P407 (F3) showed a gelation temperature of 28.8 ± 0.6 °C, which was significantly lower than that prepared at 20% *w*/*v* P407 (F1; 36.8 ± 0.3 °C). On the other hand, at fixed P407 concentration, increasing P188 concentration from 5% *w*/*v* to 10% *w*/*v* tended to elevate gelation temperature, as summarized in Table 2. The gelation temperature of F7 (40.5 ± 0.8 °C), prepared at 10% *w*/*v* P188 was remarkably higher than that of F1 (36.8 ± 0.3 °C), prepared at 5% *w*/*v* P188. Poloxamers are triblock copolymers composed of a core poly (propylene oxide) hydrophobic portion (PPO) and two poly (ethylene oxide) hydrophilic parts (PEO) [23]. Many reports have revealed that gelation properties of poloxamers relies on the ratio between the hydrophobic and hydrophilic sub-units (PPO/PEO) in the polymer chain [23,24,25]. Reducing PPO/PEO ratio, by either decreasing P407 concentration or increasing P188 concentration, resulted in a significant rise (*p* < 0.05) in the gelation temperature of in situ gel formulation. This might be accounted for the abundant hydrogen bonds between the comparatively hydrophilic PEO blocks and water, which raises the energy necessary to break down the hydrogen bonds between water and PEO blocks and, as a result, raises the sol-to-gel transition temperature [26]. Similar results were reported by Cao et al. who highlighted the impact of increasing poloxamer 407 concentrations in promoting gel formation at lower temperatures for poloxamer-based azithromycin in situ gel formulations [27]. 

#### 2.2.5. In Vitro Muco-Adhesion Force

**Figure 1:**
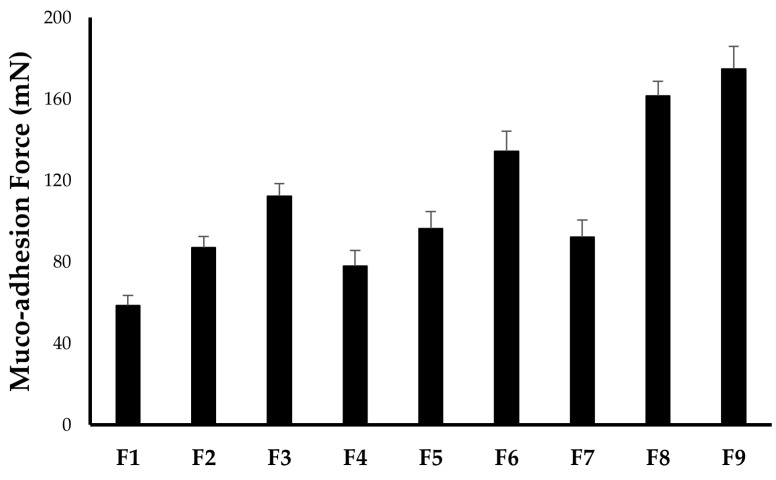
In vitro muco-adhesion force of various CS-ISG formulations. The data represent the mean ± SD of three independent experiments.

Muco-adhesion force is the force with which the formulation binds to the corneal surface. It is one of the essential parameters for ocular ISGs formulation since the ability of ISGs to increase pre-corneal residence does not depend only on the ability to be converted into gel after instillation into the eye but on the muco-adhesion power of the formed ISGs as well. Generally, polymers with good muco-adhesion properties will increase the muco-adhesion force, prolong the pre-corneal residence time of the formulation, and thereby, enhance the overall ocular bioavailability [28,29]. As summarized in Table 2 and Figure 1, the muco-adhesion force of all ISGs formulations fluctuates from 58.7 ± 4.9 mN (F1) to 174.8 ± 11.2 mN (F9). It was evident that increasing either P407 or P188 concentration could result in a proportional enhancement in the muco-adhesion force between the prepared ISGs formulations and corneal surface, presumably, owing to the elevated number of co-polymer chains penetrating glycoprotein chains per unit volume of mucin [30].

#### 2.2.6. Rheological Studies

Rheological properties play a key role in the formulation of in situ gelling solutions. In general, ocular in situ gels should have a viscosity that permits easy instillation into the eye and a rapid transition from sol to gel upon instillation [31]. Herein, the rheological properties of all CS-ISG formulations were examined as a function of temperature. The average results of the viscosity of the prepared ISGs before (at 25 °C) and after (35 °C) gelling are tabulated in Table 2. As presented in Table 2, all CS-ISG formulations had low viscosities at ambient temperature (25 °C), but when the temperature was raised to 37 °C, a significant rise in the viscosity of all formulations was observed, presumably, because of the thermosensitive in situ gelling property of these polymers systems. In addition, it was obvious that, for all tested formulations, there was a remarkable increase in the viscosity with increments in the concentrations of either P407 or P188. For instance, at fixed P188 concentration, the viscosity of CS-ISG formulation prepared at 25% *w*/*v* P407 (F3; 112.8 ± 9.3 cp) was significantly higher than that prepared at 20% *w*/*v* P407 (F1; 58.6 ± 4.2 cp). In the same context, increasing co-polymer (P188) concentration was associated with a pronounced increase in CS-ISGs viscosities. For example, at fixed P407 concentration, the viscosity of CS-ISG formulation prepared at 10% *w*/*v* P188 (F9; 164.3 ± 12.0 cp) was obviously higher than that prepared at 5% *w*/*v* P188 (F3; 112.8 ± 9.3 cp). The same trend of a mutual increase in CS-ISGs viscosities with increasing either P407 or P188 concentrations was observed at 35 °C. This increase in viscosity might be explained by the interaction of the co-polymer (P188) with the micellar entanglement of P407, which could cause the creation of stronger bonds and hence an increase in formulation viscosity. Of note, viscosity results revealed that F1, F2, F4 and F7 met the requirement of ophthalmic in situ gel viscosity 5–100 cPs at room temperature [32]. Nevertheless, F1, F4 and F7 did not fulfill the criterion of having appropriate gelation temperature for ophthalmic application; all these formulations showed TG values greater than pre-corneal temperature (35 °C). 

Based on various characterization parameters, particularly, gelation temperature and viscosity measurements, F2 was selected for further studies since formula F1, F4, F5, F7 and F8 had high gelation temperature (>35 °C), which would hinder their transformation into gel state at corneal temperature. Whilst F3, F6, and F9 had relatively higher viscosity values at room temperature (>100 cPs) that might hinder proper in situ gel application. By contrary, the selected formula (F2) has good gelation capacity at body temperature, appropriate viscosity at 25 °C that would ease its ocular application, and relatively higher viscosity at 37 °C, which would promote prolonged ocular residence of the formulation post its instillation into eyes.

### 2.3. In Vitro Release Studies

**Figure 2:**
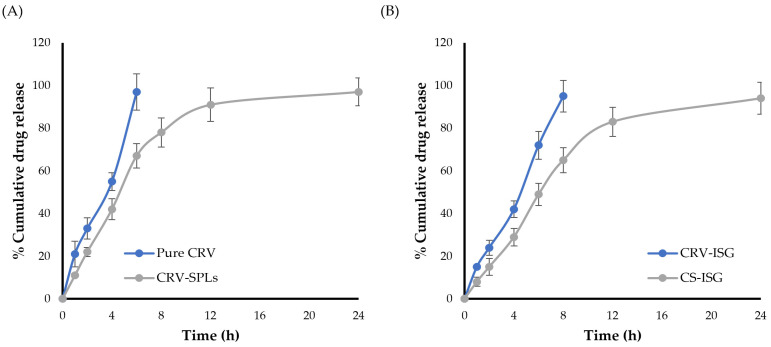
In vitro release profile of CRV from (**A**) SPLs in comparison with pure CRV and (**B**) Spanlastic laden in situ gel (F2) in comparison with CRV-loaded in situ gel (CRV-ISG). The data represent the mean ± SD of three independent experiments.

The in vitro release profiles of CRV from different formulations was investigated using STF (pH 7.4) as a dissolution medium. As depicted in Figure 2A, it was clear that entrapping CRV within spanlastic (SPLs) system greatly slowed drug release. For CRV suspension, ~30% of CRV was released after 2 h and ~95% after 6 h. On the other hand, CRV-SPLs demonstrated biphasic release, with ~40% of CRV was rapidly released in the first 4 h, presumably owing to surface-adsorbed free CRV, followed by continuous release from the vesicle core for up to 24 h. Such slower release pattern of CRV from spanlastic formulation was ascribed to the entrapment of CRV within vesicular system (spanlastics), which is known to operate as a reservoir that slows down drug release, resulting in a prolonged release profile. 

The in vitro release profiles of selected CS-ISG formulation (F2), compared to CRV-loaded in situ gel (CRV-ISG) were graphically illustrated in Figure 2B. In vitro release results inferred that incorporating CRV-SPLs into ISGs had significantly sustained drug release, compared to plain CRV-ISG. The percentage cumulative CRV released from CS-ISG formulation (F2) in 8 h was ~70%, compared to 95% for plain CRV-ISG. This slower drug release from CS-ISG formulation (2) might be related to the dual action of including the vesicular system (CRV-SPLs) within in situ gelling system.

### 2.4. Ex Vivo Corneal Permeability

**Figure 3:**
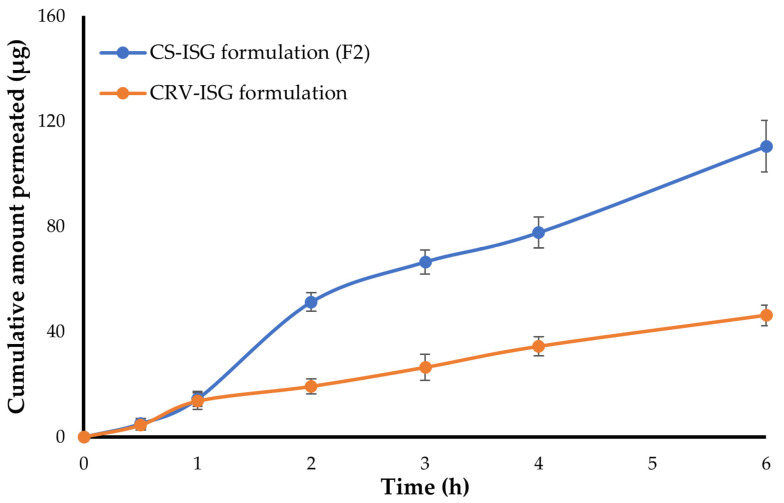
Transcorneal permeability of CRV across rabbit cornea from CRV-loaded in situ gel (CRV-ISG) and optimized CS-ISG (F2) (mean ± SD, n = 3).

Ex-vivo drug permeation of plain CRV-ISG and CS-ISG formulation (F2) was conducted using goat corneal membrane since it simulates the condition of the human corneal membrane [33]. Figure 3 depicts the cumulative amount of CRV permeated through the cornea membrane from both CRV-ISG and CS-ISG formulation. Figure 3 inferred that CRV release from either CRV-ISG and CS-ISG formulation (F2) was comparable in the first hour. Following that, an increase in CRV permeation was observed with CS-ISG formulation (F2) when compared to plain CRV-ISG formulation. The cumulative amount of CRV permeated after 6 h (Q_6h_) from CS-ISG formulation (F2) was 110.4 ± 9.8 μg, which was ~3 times higher than that of plain CRV-ISG (Q_6h_ 46.2 ± 3.9 μg). The remarkable increase in drug permeation from CS-ISG formulation might be ascribed primarily to the encapsulation of the drug with nano-size vesicles (spanlastics) with superior corneal penetration properties. Similar results were reported by Hassan et al. [6] who underscored the potential of entrapping carvedilol within cationic nanoparticles (leciplex) for enhancing the transcorneal penetration of CRV compared to plain drug solution. 

In addition, transcorneal permeation parameters for both plain CRV-ISG and CS-ISG formulation (F2) were calculated (Table 3). As summarized in Table 3, CS-ISG formulation (F2) showed greater steady state transcorneal flux (J_ss_ 22.37 ± 2.1 μg/h) compared to plain CRV-ISG (J_ss_ 8.38 ± 1.1 μg/h). Interestingly, F2 triggered a considerable enhancement in CRV apparent permeation coefficient (P_app_ 6.39 × 10^−6^ cm/s) compared to plain CRV-ISG (P_app_ 2.67 × 10^−6^ cm/s). This might be ascribed to the presence of span 60 and EA in the spanlastics, which would promote higher drug permeation through corneal membrane [34]. Collectively, it is possible to infer that entrapment of CRV within SPLs greatly improved CRV corneal penetration over the plain ISG formulation.

### 2.5. In Vitro Stability Study

The optimized CS-ISG formulation (F2) was subjected to stability study following storage at 4 °C for 8 weeks. Visual appearance, pH, drug content and gelling capability were examined. Based on a 2-month investigation, there is little difference in visual appearance, pH, drug content and gelling capability, inferring that the formulation is stable (Table 4). 

### 2.6. In Vivo Pharmacokinetic Study

**Figure 4:**
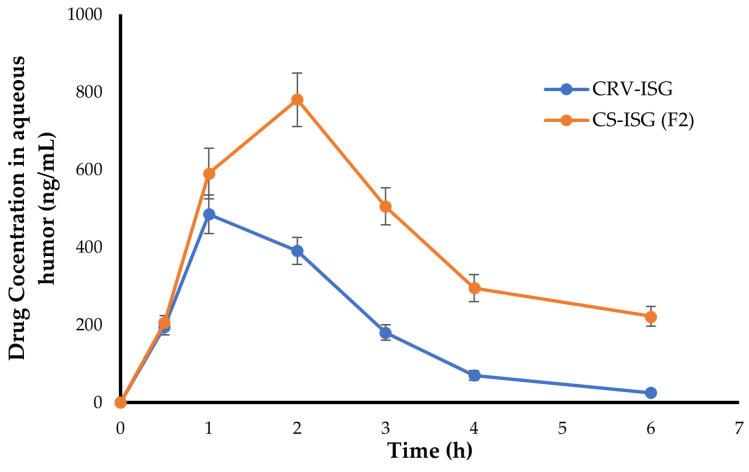
Carvedilol concentration in the aqueous humor following single ocular instillation of various formulations. The data represent mean ± SD (n = 6).

In vivo pharmacokinetic study was performed to estimate the ocular bioavailability, based on calculating the amount of CRV penetrated to the aqueous humor of rabbit eyes, following a single instillation of either plain CRV-ISG and CS-ISG formulation (F2). Non-compartment model analysis was implemented to calculate several pharmacokinetic parameters such as C_max_, t_max_, and AUC from a graph drawn between CRV concentrations (ng/mL) in aqueous humor and time [35]. As shown in Figure 4, CRV levels in aqueous humor were elevated rapidly within 1 h post instillation of either plain CRV-ISG and CS-ISG formulation (F2), indicating relatively rapid onset of action. Nevertheless, for plain CRV-ISG, drug levels in aqueous humor declined rapidly, where very low concentrations of CRV were detected in aqueous humor at 4 h post instillation. On the other hand, F2 showed higher drug levels in the aqueous humor for an extended period of time, suggesting a dramatic increase in drug penetration through corneal membrane.

The computed pharmacokinetic parameters for both plain CRV-ISG and CS-ISG formulation (F2) were tabulated in Table 5. As depicted in Table 5, plain CRV-ISG showed t_max_ of 1 h, which was remarkably shorter than that observed with CS-ISG formulation (F2; t_max_ = 2 h). This delayed t_max_ of F2 compared to plain CRV-ISG might be accounted to the entrapment of CRV into spanlastic vesicles, which might pose an additional diffusion barrier for drug release into aqueous humor. Nevertheless, it was obvious that F2 had a significantly higher peak concentration (C_max_ 781.4 ± 69.4 ng/mL) and greater AUC_0–6h_ (2494.5 ± 113.7 ng·h/mL) compared to plain CRV-ISG (C_max_; 485.7 ± 52.9 ng/mL and AUC_0–6h_ 1161.3 ± 98.6 ng·h/mL). These results are consistent with ex vivo permeation data, in which, the flux (J_ss_) and apparent permeability coefficient (P_app_) were considerably higher in F2 (Figure 3 and Table 4). Furthermore, the mean residence time (MRT) of F2 was 4.11 ± 0.5 h, which was longer than that of CRV-ISG (MRT = 2.15 ± 0.3 h). This increase in MRT for F2 could be ascribed to the gradual and prolonged release of CRV from spanlastic vesicles. Collectively, our results underscored the potential of CRV-loaded spanlastic vesicles to augment the ocular bioavailability of CRV. These findings are consistent with previous research on the effect of drug encapsulation into nanoparticulate systems on drug ocular pharmacokinetics. For instance, Huang et al. discovered that, as compared to commercial timolol eye drops, cubosomes had the ability to sustain timolol release, and thereby, foster its retention in the aqueous humor and the anterior segment of eye [36]. Similarly, Ban et al. accentuated the efficacy of charged lipid nanoparticle to extend dexamethasone retention time and to boost its permeation through the cornea, resulting in higher ocular bioavailability when compared to dexamethasone solution [37].

### 2.7. In Vivo Pharmacodynamic Study

**Figure 5:**
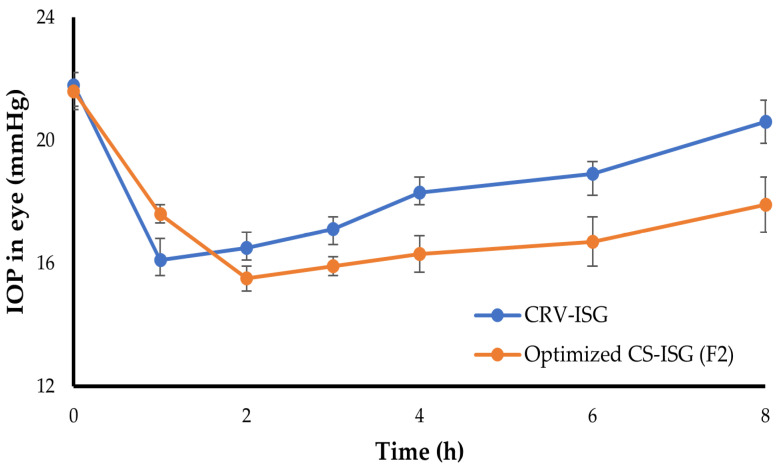
IOP lowering effect CRV-ISG and CS-ISG (F2). The data represent mean ± SD (n = 6).

The in vivo efficacy of the optimized CS-ISG formulation (F2) on reducing IOP was investigated and compared to that of plain CRV-ISG. The change in IOP from baseline with time following ocular instillation of either optimized F2 formula or plain CRV-ISG was plot in Figure 5. As shown in Figure 5, plain CRV-ISG succeeded to elicit a rapid drop in IOP (16.1 ± 0.5 mmHg) after one hour following ocular administration that lasted for three hours, following which IOP progressively increased to its initial value (20.6 ± 0.7 mmHg) at eight hours. On the other hand, in comparison to plain CRV-ISG, the optimized F2 formula triggered a substantial decrease in IOP readings after 2 h, with a maximum reduction of 15.1 ± 0.4 mmHg. This IOP lowering activity was maintained for up to 8 h post F2 instillation, indicating a sustained action of F2. The average IOP at 8 h post F2 instillation was 17.9 ± 0.9 mmHg, compared to 21.6 ± 0.6 mmHg for control eye. These results suggest that the inclusion of CRV-SPLs into ISGs formulation would sustain drug release for more prolonged time than plain CRV-ISG, and subsequently, the instilled dose could be decreased. The double-layered structure of spanlastic, gel viscosity, and gel matrix structure all contributed to the prolonged and sustained impact [38]. Similar results were stated by Leonardi et al. who investigated the IOP lowering activity of cationic solid lipid nanoparticle encapsulating melatonin. They revealed that solid lipid nanoparticle entrapping melatonin had a superior IOP lowering activity that lasted for 24 h after instillation, compared to that of free drug, which exerted its effect for only 4 h post ocular application [39]. 

### 2.8. Ocular Irritation

The Draize rabbit eye test was adopted to scrutinize the possible irritation potential of optimized F2 formula. In this study, ocular irritation was studied after ocular instillation of CRV-ISG and optimized CS-ISG formulations. Both treated groups showed no symptoms of ocular irritation such as tears, redness, or edema during the test (Table S1). These findings ruled out the irritating potential of the test formulations.

## 3. Conclusions

In this study, we explored the influence of incorporating carvedilol-loaded spanlastics into in situ gelling system on the anti-glaucoma action of carvedilol. Thermosensitive in situ gel was prepared with a blend of two poloxamer analogs; 22% *w*/*v* poloxamer 407 and 5% *w*/*v* poloxamer 188. Incorporating CRV-loaded spanlastics within poloxamer-based in situ gel provided a dual action on sustaining drug release and prolonging the corneal retention time. In addition, ex vivo permeation studies demonstrated that the optimized spanlastic-laden in situ gel formulation (F2) significantly enhanced CRV permeation across the rabbit cornea by a 2.4-fold compared to plain CRV-ISG formulation. Most importantly, in vivo studies verified that incorporating CRV-loaded spanlastics within poloxamer-based in situ gel triggered a 2-fold increase in the AUC of optimized formula (F2), compared to plain CRV-ISG formulation. This enhancement in CRV ocular bioavailability was synchronized with a superior IOP lowering potential of optimized formula (F2). Collectively, spanlastic laden in situ gel might represent a promising alternative to conventional dosage forms for promoting efficient corneal delivery of anti-glaucoma drugs.

## 4. Materials and Methods

### 4.1. Materials

Carvedilol (CRV) was generously obtained from SAGA Pharmaceutical Company (Cairo, Egypt). Brij 97, poloxamer 407, poloxamer 188 and Span 60 were provided by Sigma Aldrich (St. Louis, MO, USA). All other used chemicals were of analytical grade.

### 4.2. Formulation of Carvedilol-Loaded Spanlastics (CRV-SPLs)

Carvedilol (CRV)-loaded spanlastics (CRV-SPLs) were fabricated by the ethanol injection method, as described previously [21]. Span 60 was adopted as the main vesicle forming component, while Brij 97 was used as an edge activator (EA) at Span 60:EA weight ratio of 90:10. To prepare CRV-loaded SPLs, CRV (62.5 mg) and a definite weight of Span 60 were dissolved in 5 mL ethanol. The ethanolic solution was then added dropwise to 15 mL of a preheated Brij 97 aqueous solution (70 °C). The dispersion was continuously stirred on a magnetic agitator (Jenway 1000, Jenway, UK) till the formation of milky spanlastic dispersion. The resultant dispersion was sonicated for 5 min to minimize the particle size. Finally, the obtained dispersion was stored at 4 °C until being used in further experiments.

### 4.3. Incorporation of CRV-SPLs into In Situ Gels (CS-ISGs) 

Poloxamer-based hydrogel containing CRV-SPLs equivalent to 0.5% *w*/*w* of the drug were fabricated by the cold method [40] using definite concentrations of Poloxamer 407 (P407) and Poloxamer 188 (P 188) as summarized in Table 1. Briefly, definite concentrations of both P407 and P188 were mixed together and were dissolved in specific volumes of deionized water at 4 ± 1 °C. The mixture was stirred continuously overnight until a clear homogenous solution without lumps was obtained. Finally, an accurately weighed amount of CRV-SPLs was uniformly dispersed in the preformed poloxamer-based hydrogel and stored overnight in a refrigerator to exclude any entrapped air bubbles for further examinations. 

### 4.4. Characterization of CRV-SPLs-Loaded In Situ Gels (CS-ISGs) 

#### 4.4.1. Visual Appearance

Clarity and homogeneity of the prepared CS-ISGs were observed by visual inspection of different formulations against a black and white background. 

#### 4.4.2. pH 

pH meter (CG820 Schott Geräte,, Gerbershausen, Germany) was adopted for measuring the pH values of various formulation. 

#### 4.4.3. Drug Content 

0.5 g of CS-ISGs were dissolved in ethanol using sonication to thoroughly lyse the vesicles then filtered through a 0.45 m milipore filter. The filtrate was suitably diluted, and the drug content was finally quantified spectrophotometrically at 242 nm using Ultraviolet–visible (UV–Vis) spectrophotometer (Shimadzu, Kyoto, Japan). The drug content (%) was calculated using the following formula:

$$Drug\;content\;\left(\%\right)=\frac{Actual\;amount\;of\;CRV}{Theoretical\;amount\;of\;CRV}\times 100$$

#### 4.4.4. Determination of Gelation Temperature

Gelation temperature (T_G_) was assessed by an inversion method [41]. Briefly, 1 mL of each CS-ISG formulation was placed into a 2 mL Eppendorf tube and allowed to equilibrate for 5 min at room temperature. The tubes were then placed in a thermomixer (Eppendorf ThermoMixer^®^ C, Enfield, CT, USA) that had previously adjusted at 20 °C and subjected to a temperature rise of 1 °C every 2 min. The temperature at which no movement into the liquid was detected upon tilting up the tubes at 90° is referred to as the sol-gel transition temperature. 

#### 4.4.5. Rheological Studies

Brookfield viscometer model DVII (Haake Inc., Osterode am Harz, Germany) was used for viscosity measurements of different CS-ISG formulations before and after gelation (at 25 °C and 37 °C). Briefly, one gram of gel under investigation was put in the sample holder, and spindle no. 4 was lowered perpendicularly into it. The spindle was rotated at a constant speed of 100 rpm, and all measures were done in triplicates.

#### 4.4.6. Measurement of Muco-Adhesion Force

The muco-adhesion force was determined using the modified analytical two-pan balance [28]. Freshly excised goat cornea was obtained from a local slaughterhouse. The cornea was excised from the ocular tissue and rinsed several times with cold PBS (pH 7.4) to get off any protein debris. Two same glass slides were used; one was fitted on the lower pan using double-sided adhesive tape, and the other was fitted on a table bench. Two same pieces of the cornea (2.5 cm^2^) were adhered to each slide using glue. A very thin layer of a specific weight (0.5 gm) of each CS-ISG formulation was applied between corneal tissues. A preload of 5 gm was applied over the balance pan above glass slides for 30 s and then removed to ensure intimate contact between the excised cornea and the ISGs formulation. Increasing amounts of water were added in the second pan until the slides detached from each other and the water weight that cause complete detachment was recorded. The force of adhesion (N), defined as the minimum weight needed to detach the cornea from the formulation, was calculated using the following equation [42]:

$$N=\frac{m\;\cdot \;g}{1000}$$

where (m) is the weight in grams of water needed to detach the CS-ISGs formulation away from the cornea; (g) is the gravitational acceleration (9.81 m/s^2^). 

### 4.5. In Vitro Release Study

In vitro release of CRV from different CS-ISGs formulations was performed using a modified Franz diffusion cell, employing freshly prepared simulated tear fluid (STF; pH 7.4) as the release medium. In brief, a specific volume of each formula corresponding to 5 mg of CRV was transferred to the donor chamber. The donor chamber was then suspended in 250 mL of the release medium placed in the receptor compartment and was kept at 37 ± 0.5 °C and constantly agitated at 100 rpm. At predetermined time intervals, 1 mL samples were collected and replaced with an equal volume of fresh medium. The collected aliquot samples were diluted and spectrophotometrically analyzed at 242 nm to quantify the amount of drug released. 

### 4.6. Ex Vivo Corneal Permeability Study

Ex vivo corneal permeability across freshly excised rabbit cornea was investigated using the membrane diffusion method [43]. Briefly, the excised cornea was sandwiched between the donor and receptor compartments of Franz diffusion cell. Both free CRV solution and selected CS-ISG formulation (equal to 1 mg CRV) were applied to the corneal epithelium in the donor compartment. 25 mL of fresh STF (pH 7.4) was used as the receptor medium. At predetermined time intervals, 1 mL aliquots were removed and replaced with fresh medium. The samples were spectrophotometrically analyzed at 242 nm to quantify drug content in each sample. The cumulative amount of CRV permeated through corneal membrane per unit area was plotted versus time (h). Drug flux across cornea was determined from the slope of the linear part of the curve, while the apparent permeability coefficient was calculated using the following formula:

$$P_{app}=\frac{∆Q}{∆t}\;.\;\frac{1}{\;3600\times {A\times C}_{ο}}$$

where, ∆Q/∆t is the cumulative amount of drug permeated across the cornea over time t, A is the exposed corneal surface area (0.8 cm^2^), and C_ο_ is the initial drug concentration in donor chamber.

### 4.7. Stability Studies

The stability studies for the optimized CS-ISG formulation were carried out by storing the optimized formula at 4 °C for 8 weeks and then the formula was assessed for visual appearance, pH, drug content and gelling capability.

### 4.8. In Vivo Experiments

#### 4.8.1. In Vivo Pharmacokinetics

In vivo pharmacokinetic study was conducted on male albino rabbits (2–2.5 kg). The study protocol was reviewed by the Animal Ethics Committee, Prince Sattam Bin Abdulaziz University, Al-Kharj, KSA (approval number: 048/2022). In this study, the animals were categorized into two groups; the first group was treated with 50 μL of CS-ISG formulation (5 mg CRV/mL), while the other group was treated with 50 μL of CRV ophthalmic suspension (0.5% *w*/*v*). Animal eyelids were lightly closed for 1 min to permit better contact of drug with the corneal membrane. Prior to aqueous humor withdrawal, rabbits were anaesthetized with sodium phenobarbital (30 mg/kg), and 100 μL samples of aqueous humor were obtained at 0.5, 1, 2, 4, and 6 h using a 29-gauge insulin syringe needle. The samples were mixed with 500 μL of methanol to precipitate protein, followed by centrifugation at 5000 rpm for 15 min. The concentration of CRV in the supernatant was quantified using an HPLC system equipped with a UV detector (Shimadzu, Tokyo, Japan) at 240 nm and a Hypersil^®^ C-18 column (150 mm × 4.6 mm, 5 μm). The column was eluted with a mobile phase consisting of KH_2_PSO_4_:acetonitrile (50:50 *v*/*v*), adjusted to pH 3.0 with dilute orthophosphoric acid solution. The flow rate was 1 mL/min and the injection volume was 20 μL. The pharmacokinetic parameters were determined using a PKSolver 2.0 software.

#### 4.8.2. Pharmacodynamic Study

In vivo pharmacodynamic study was conducted on male albino rabbits, with an average IOP value of 21.6 mmHg. Twelve rabbits were divided into two groups: Group I was instilled with 50 μL of CRV suspension (0.5% *w*/*v*), while Group II was instilled with 50 μL of CS-ISG formulation (5 mg CRV/mL), into the left eye. The right eye received 50 μL physiological saline and served as control. At predetermined time points post treatments (0, 0.5, 1, 2, 3, 4, 5, and 6 h), the IOP was measured under surface anesthesia with 0.2% lidocaine using a tonometer (Riester, Jungingen, Germany). 

#### 4.8.3. Assessment of Ocular Irritancy of CS-ISG Formulation

Male albino rabbits (2 groups of 3 animals in each) were employed to examine the ocular tolerability of the formulated CS-ISG formulation. The animals were inspected for any signs of irritation (redness, inflammation, or increased tear production) upon ocular application. A 50 μL aliquot of CS-ISG formulation or an equivalent concentration of CRV-ISG were ocularly applied into the left eye’s conjunctival sac, while the contralateral eye served as a control and received no treatment. Direct visual inspection using a slit lamp was used to examine both eyes of the rabbits for any signs of irritation.

## Figures and Tables

**Table 1:** Composition of various CRV-SPLs-loaded in situ gels (CS-ISGs) formulations.

Formula	Poloxamer 407	Poloxamer 188
F1	20	5
F2	22	5
F3	25	5
F4	20	7.5
F5	22	7.5
F6	25	7.5
F7	20	10
F8	22	10
F9	25	10

**Table 2:** Physicochemical properties of different CS-ISG formulations.

Formula	pH	Drug Content (%)	Gelation Temperature (T_G_; °C)	Muco-Adhesion Force (mN)	Viscosity (cp)	
					At 25 °C	At 37 °C
F1	6.48 ± 0.04	99.3 ± 0.4	36.8 ± 0.3	58.7 ± 4.9	58.6 ± 4.2	276.3 ± 13.8
F2	6.94 ± 0.03	99.1 ± 0.5	32.4 ± 0.5	87.2 ± 5.2	83.6 ± 5.9	369.3 ± 10.9
F3	7.13 ± 0.07	98.6 ± 0.7	28.8 ± 0.6	112.3 ± 6.1	112.8 ± 9.3	424.3 ± 13.7
F4	6.71 ± 0.06	98.4 ± 0.4	37.9 ± 0.5	78.1 ± 7.6	76.3 ± 4.4	303.5 ± 10.8
F5	7.03 ± 0.05	98.3 ± 0.6	34.9 ± 0.7	96.4 ± 8.3	103.3 ± 6.1	363.7 ± 18.8
F6	7.29 ± 0.08	97.9 ± 0.9	31.5 ± 0.4	134.5 ± 9.7	146.4 ± 11.5	485.3 ± 43.9
F7	6.87 ± 0.08	98.2 ± 0.7	40.5 ± 0.8	92.3 ± 8.2	96.1 ± 6.5	362.7 ± 11.7
F8	7.18 ± 0.06	97.8 ± 1.1	36.9 ± 0.9	161.5 ± 7.3	114.7 ± 9.6	454.9 ± 11.4
F9	7.32 ± 0.05	97.3 ± 0.9	33.6 ± 0.7	174.8 ± 11.2	164.3 ± 12.0	549.8 ± 15.2

All data represents mean ± SD. (n = 3).

**Table 3:** Ex vivo permeation parameters of different CRV formulations.

Formula	J_ss_ (μg/h)	P_app_ (cm/s) × 10^−6^	Q_6h_ (μg)
Plain CRV-ISG	8.38 ± 0.95	2.67	46.2 ± 3.9
CS-ISG formulation (F2)	22.37 ± 2.24	6.39	110.4 ± 9.8

**Table 4:** Stability study of optimized CS-ISG formulation (F2).

Time	Visual Appearance	pH	Drug Content	Gelling Capacity
0	Clear	6.94 ± 0.03	99.1 ± 0.5	+++
4th week	Clear	7.05 ± 0.10	98.3 ± 1.0	+++
8th week	Clear	7.21 ± 0.09	97.5 ± 1.3	+++

Data represent mean ± SD. (n = 3). +++ Gelation immediately, remains for extended period.

**Table 5:** Pharmacokinetic parameters of different carvedilol formulations in aqueous humor.

Pharmacokinetic Parameter	CRV-ISG	CS-ISG (F2)
C_max_ (ng/mL)	485.7 ± 52.9	781.4 ± 69.4
t_max_ (h)	1	2
t_1/2(h)_	1.01 ± 0.2	2.21 ± 0.4
AUC_0–6h_ (ng·h/mL)	1161.3 ± 98.6	2494.5 ± 113.7
MRT (h)	2.15 ± 0.3	4.11 ± 0.5

## Data Availability

Data are contained within the article or Supplementary Materials.

## Supplementary Materials

The following supporting information can be downloaded at: https://www.mdpi.com/article/10.3390/gels9120952/s1, Table S1: Grading of ocular irritation by Draize irritation test in rabbits.

## References

[1] Weinreb R.N., Aung T., Medeiros F.A. (2014). The pathophysiology and treatment of glaucoma: A review. JAMA.

[2] Alamri A., Bakri K.A., Alqarni S.M., Alosaimi M.N., Alshehri E.Y., Alshahrani M.S. (2022). Knowledge and awareness of glaucoma in a population of Abha, Southern Saudi Arabia. J. Fam. Med. Prim. Care.

[3] Sharif N.A. (2021). Therapeutic Drugs and Devices for Tackling Ocular Hypertension and Glaucoma, and Need for Neuroprotection and Cytoprotective Therapies. Front. Pharmacol..

[4] Öztürk K., Arslan F.B., Öztürk S.C., Çalış S. (2022). Mixed micelles formulation for carvedilol delivery: In-vitro characterization and in-vivo evaluation. Int. J. Pharm..

[5] Abdelmonem R., Elhabal S.F., Abdelmalak N.S., El-Nabarawi M.A., Teaima M.H. (2021). Formulation and Characterization of Acetazolamide/Carvedilol Niosomal Gel for Glaucoma Treatment: In Vitro, and In Vivo Study. Pharmaceutics.

[6] Hassan D.H., Abdelmonem R., Abdellatif M.M. (2018). Formulation and Characterization of Carvedilol Leciplex for Glaucoma Treatment: In-Vitro, Ex-Vivo and In-Vivo Study. Pharmaceutics.

[7] Irimia T., Ghica M.V., Popa L., Anuţa V., Arsene A.L., Dinu-Pîrvu C.E. (2018). Strategies for Improving Ocular Drug Bioavailability and Corneal Wound Healing with Chitosan-Based Delivery Systems. Polymers.

[8] Rawat P.S., Ravi P.R., Mir S.I., Khan M.S., Kathuria H., Katnapally P., Bhatnagar U. (2023). Design, Characterization and Pharmacokinetic&ndash;Pharmacodynamic Evaluation of Poloxamer and Kappa-Carrageenan-Based Dual-Responsive In Situ Gel of Nebivolol for Treatment of Open-Angle Glaucoma. Pharmaceutics.

[9] null A., Ali J., Fazil M., Qumbar M., Khan N., Ali A. (2016). Colloidal drug delivery system: Amplify the ocular delivery. Drug Deliv..

[10] Ahmed S., Amin M.M., Sayed S. (2023). Ocular Drug Delivery: A Comprehensive Review. AAPS PharmSciTech.

[11] Silva B., São Braz B., Delgado E., Gonçalves L. (2021). Colloidal nanosystems with mucoadhesive properties designed for ocular topical delivery. Int. J. Pharm..

[12] Shukr M.H., Ismail S., El-Hossary G.G., El-Shazly A.H. (2022). Spanlastics nanovesicular ocular insert as a novel ocular delivery of travoprost: Optimization using Box–Behnken design and in vivo evaluation. J. Liposome Res..

[13] Kakkar S., Kaur I.P. (2011). Spanlastics—A novel nanovesicular carrier system for ocular delivery. Int. J. Pharm..

[14] Rathod S., Arya S., Shukla R., Ray D., Aswal V.K., Bahadur P., Tiwari S. (2021). Investigations on the role of edge activator upon structural transitions in Span vesicles. Colloids Surf. A Physicochem. Eng. Asp..

[15] Abdelbari M.A., El-Mancy S.S., Elshafeey A.H., Abdelbary A.A. (2021). Implementing Spanlastics for Improving the Ocular Delivery of Clotrimazole: In vitro Characterization, Ex vivo Permeability, Microbiological Assessment and In vivo Safety Study. Int. J. Nanomed..

[16] Shymborska Y., Budkowski A., Raczkowska J., Donchak V., Melnyk Y., Vasiichuk V., Stetsyshyn Y. (2023). Switching it Up: The Promise of Stimuli-Responsive Polymer Systems in Biomedical Science. Chem. Rec..

[17] Nagai N., Minami M., Deguchi S., Otake H., Sasaki H., Yamamoto N. (2020). An in situ Gelling System Based on Methylcellulose and Tranilast Solid Nanoparticles Enhances Ocular Residence Time and Drug Absorption Into the Cornea and Conjunctiva. Front. Bioeng. Biotechnol..

[18] Wu Y., Liu Y., Li X., Kebebe D., Zhang B., Ren J., Lu J., Li J., Du S., Liu Z. (2019). Research progress of in-situ gelling ophthalmic drug delivery system. Asian J. Pharm. Sci..

[19] Abbas M.N., Khan S.A., Sadozai S.K., Khalil I.A., Anter A., Fouly M.E., Osman A.H., Kazi M. (2022). Nanoparticles Loaded Thermoresponsive In Situ Gel for Ocular Antibiotic Delivery against Bacterial Keratitis. Polymers.

[20] Aldawsari M.F., Moglad E.H., Alotaibi H.F., Alkahtani H.M., Khafagy E.-S. (2023). Ophthalmic Bimatoprost-Loaded Niosomal In Situ Gel: Preparation, Optimization, and In Vivo Pharmacodynamics Study. Polymers.

[21] Sallam N.M., Sanad R.A.B., Ahmed M.M., Khafagy E.L.S., Ghorab M., Gad S. (2021). Impact of the mucoadhesive lyophilized wafer loaded with novel carvedilol nano-spanlastics on biochemical markers in the heart of spontaneously hypertensive rat models. Drug Deliv. Transl. Res..

[22] Abelson M.B., Udell I.J., Weston J.H. (1981). Normal human tear pH by direct measurement. Arch. Ophthalmol..

[23] Zarrintaj P., Ramsey J.D., Samadi A., Atoufi Z., Yazdi M.K., Ganjali M.R., Amirabad L.M., Zangene E., Farokhi M., Formela K. (2020). Poloxamer: A versatile tri-block copolymer for biomedical applications. Acta Biomater..

[24] Liu S., Bao H., Li L. (2015). Role of PPO–PEO–PPO triblock copolymers in phase transitions of a PEO–PPO–PEO triblock copolymer in aqueous solution. Eur. Polym. J..

[25] Russo E., Villa C. (2019). Poloxamer Hydrogels for Biomedical Applications. Pharmaceutics.

[26] Abdeltawab H., Svirskis D., Hill A.G., Sharma M. (2022). Increasing the Hydrophobic Component of Poloxamers and the Inclusion of Salt Extend the Release of Bupivacaine from Injectable In Situ Gels, While Common Polymer Additives Have Little Effect. Gels.

[27] Cao F., Zhang X., Ping Q. (2010). New method for ophthalmic delivery of azithromycin by poloxamer/carbopol-based in situ gelling system. Drug Deliv..

[28] Al-Kassas R.S., El-Khatib M.M. (2009). Ophthalmic controlled release in situ gelling systems for ciprofloxacin based on polymeric carriers. Drug Deliv..

[29] Al Khateb K., Ozhmukhametova E.K., Mussin M.N., Seilkhanov S.K., Rakhypbekov T.K., Lau W.M., Khutoryanskiy V.V. (2016). In situ gelling systems based on Pluronic F127/Pluronic F68 formulations for ocular drug delivery. Int. J. Pharm..

[30] Jaipal A., Pandey M.M., Charde S.Y., Raut P.P., Prasanth K.V., Prasad R.G. (2015). Effect of HPMC and mannitol on drug release and bioadhesion behavior of buccal discs of buspirone hydrochloride: In-vitro and in-vivo pharmacokinetic studies. Saudi Pharm. J..

[31] Gupta H., Jain S., Mathur R., Mishra P., Mishra A.K., Velpandian T. (2007). Sustained ocular drug delivery from a temperature and pH triggered novel in situ gel system. Drug Deliv..

[32] Kurniawansyah I.S., Rusdiana T., Sopyan I., Ramoko H., Wahab H.A., Subarnas A. (2020). In situ ophthalmic gel forming systems of poloxamer 407 and hydroxypropyl methyl cellulose mixtures for sustained ocular delivery of chloramphenicole: Optimization study by factorial design. Heliyon.

[33] Ranch K.M., Maulvi F.A., Naik M.J., Koli A.R., Parikh R.K., Shah D.O. (2019). Optimization of a novel in situ gel for sustained ocular drug delivery using Box-Behnken design: In vitro, ex vivo, in vivo and human studies. Int. J. Pharm..

[34] Gilani S.J., Jumah M.N.B., Zafar A., Imam S.S., Yasir M., Khalid M., Alshehri S., Ghuneim M.M., Albohairy F.M. (2022). Formulation and Evaluation of Nano Lipid Carrier-Based Ocular Gel System: Optimization to Antibacterial Activity. Gels.

[35] Nair A.B., Shah J., Jacob S., Al-Dhubiab B.E., Sreeharsha N., Morsy M.A., Gupta S., Attimarad M., Shinu P., Venugopala K.N. (2021). Experimental design, formulation and in vivo evaluation of a novel topical in situ gel system to treat ocular infections. PLoS ONE.

[36] Huang J., Peng T., Li Y., Zhan Z., Zeng Y., Huang Y., Pan X., Wu C.Y., Wu C. (2017). Ocular Cubosome Drug Delivery System for Timolol Maleate: Preparation, Characterization, Cytotoxicity, Ex Vivo, and In Vivo Evaluation. AAPS PharmSciTech.

[37] Ban J., Zhang Y., Huang X., Deng G., Hou D., Chen Y., Lu Z. (2017). Corneal permeation properties of a charged lipid nanoparticle carrier containing dexamethasone. Int. J. Nanomed..

[38] Natarajan J.V., Ang M., Darwitan A., Chattopadhyay S., Wong T.T., Venkatraman S.S. (2012). Nanomedicine for glaucoma: Liposomes provide sustained release of latanoprost in the eye. Int. J. Nanomed..

[39] Leonardi A., Bucolo C., Drago F., Salomone S., Pignatello R. (2015). Cationic solid lipid nanoparticles enhance ocular hypotensive effect of melatonin in rabbit. Int. J. Pharm..

[40] Brambilla E., Locarno S., Gallo S., Orsini F., Pini C., Farronato M., Thomaz D.V., Lenardi C., Piazzoni M., Tartaglia G. (2022). Poloxamer-Based Hydrogel as Drug Delivery System: How Polymeric Excipients Influence the Chemical-Physical Properties. Polymers.

[41] Balasubramaniam J., Pandit J.K. (2003). Ion-activated in situ gelling systems for sustained ophthalmic delivery of ciprofloxacin hydrochloride. Drug Deliv..

[42] Ali J., Khar R., Ahuja A., Kalra R. (2002). Buccoadhesive erodible disk for treatment of oro-dental infections: Design and characterisation. Int. J. Pharm..

[43] Bíró T., Bocsik A., Jurišić Dukovski B., Gróf I., Lovrić J., Csóka I., Deli M.A., Aigner Z. (2021). New Approach in Ocular Drug Delivery: In vitro and ex vivo Investigation of Cyclodextrin-Containing, Mucoadhesive Eye Drop Formulations. Drug Des. Devel Ther..

