# Supplementary material for: Development of Carvedilol Nanoformulation-Loaded Poloxamer-Based In Situ Gel for the Management of Glaucoma
Source: Gels. 2023 Dec 4;9(12):952. doi: 10.3390/gels9120952 (PMC10742441; doi:10.3390/gels9120952)
Supplement: Supplementary file 1 [file gels-09-00952-s001.zip › gels-2730943-supplementary.pdf]

Table S1. Grading of ocular irritation by Draize irritation test in rabbits.

Conjunctiva	Control	CRV Formulations	
		CRV-ISG	Optimized CS-ISG
Degree of redness	0	0	0
Degree of swelling	0	0	0
Secretion (discharge)	0	0	0

Degree of swelling, discharge and redness were grade on scale from 0 to 3. Score 0 indicates no redness, inflammation, or excessive tearing; 1 indicates mild redness with inflammation and minimal tears; 2 indicates moderate redness with considerable inflammation and significant tearing; and 3 indicates severe redness with severe inflammation and extensive tearing.
